# Heart Transplantation in High-Risk Recipients Employing Donor Marginal Grafts Preserved With *Ex-Vivo* Perfusion

**DOI:** 10.3389/ti.2023.11089

**Published:** 2023-07-21

**Authors:** Sandro Sponga, Igor Vendramin, Jawad Salman, Veronica Ferrara, Nunzio Davide De Manna, Andrea Lechiancole, Gregor Warnecke, Andriy Dralov, Axel Haverich, Fabio Ius, Uberto Bortolotti, Ugolino Livi, Murat Avsar

**Affiliations:** ^1^ Department of Medicine, University of Udine, Udine, Italy; ^2^ Cardiothoracic Department, University Hospital of Udine, Udine, Italy; ^3^ Department of Cardiothoracic, Transplant and Vascular Surgery, Hannover Medical School, Hannover, Germany; ^4^ Department of Cardiac Surgery, Heidelberg Medical School, Heidelberg, Germany

**Keywords:** heart transplantation, normothermic machine perfusion, high-risk recipients, donor marginal grafts, *ex-vivo* heart preservation

## Abstract

Extending selection criteria to face donor organ shortage in heart transplantation (HTx) may increase the risk of mortality. *Ex-vivo* normothermic perfusion (EVP) limits ischemic time allowing assessment of graft function. We investigated the outcome of HTx in 80 high-risk recipients transplanted with marginal donor and EVP-preserved grafts, from 2016 to 2021. The recipients median age was 57 years (range, 13–75), with chronic renal failure in 61%, impaired liver function in 11% and previous cardiac surgery in 90%; 80% were mechanically supported. Median RADIAL score was 3. Mean graft ischemic time was 118 ± 25 min, “out-of-body” time 420 ± 66 min and median cardiopulmonary bypass (CPB) time 228 min (126–416). In-hospital mortality was 11% and ≥moderate primary graft dysfunction 16%. At univariable analysis, CPB time and high central venous pressure were risk factors for mortality. Actuarial survival at 1 and 3 years was 83% ± 4%, and 72% ± 7%, with a median follow-up of 16 months (range 2–43). Recipient and donor ages, pre-HTx extracorporeal life support and intra-aortic balloon pump were risk factors for late mortality. In conclusion, the use of EVP allows extension of the graft pool by recruitment of marginal donors to successfully perform HTx even in high-risk recipients.

## Graphical Abstract

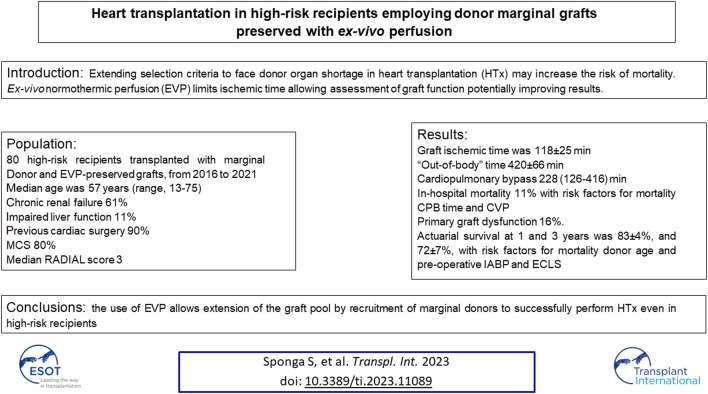

## Introduction

Orthotopic heart transplantation (HTx) is considered the gold standard treatment for patients with advanced refractory heart failure; however, while the demand is growing, the possibility to perform HTx is still limited by the chronic donor shortage [1]. Although expanding donor selection criteria could allow employment of a larger number of organs, it entails an increased risk of early and late mortality [1, 2]. This is particularly relevant in case of high-risk recipients, as those with multiple co-morbidities, a compromised clinical status or requiring pre-HTx mechanical circulatory support (MCS), in whom a more challenging surgical procedure may result in prolonged cold ischemic and bypass times, possibly jeopardizing donor heart viability [3, 4]. Based on these considerations, the use of suboptimal grafts might be a potential solution to increase the donor pool; however, whether high-risk recipients should be transplanted with marginal or regular donors is still a debated issue, which raises ethical problems requiring in some instances both physician and recipient agreement [5]. Marginal donor grafts were used in the past with traditional preservation techniques but since the results have been suboptimal especially in high risk recipients, such grafts are currently not considered for regular patients [6].

Cold static storage is the standard preservation technique of the donor graft, but it may not avoid ischemic and reperfusion injuries when preservation time exceeds 4 h [4], mainly in case of HTx with marginal grafts. In fact, it is well known how a prolonged ischemic time impacts significantly the outcome when combined with other donor and recipient variables [4].

*Ex-vivo* normothermic perfusion (EVP) is a novel procedure that, maintaining donor grafts in a beating, normothermic condition, limits ischemia-reperfusion injuries, allowing potential recovery of suboptimal organs, also favoring recruitment of longer-distance donors. Moreover, during graft transportation, EVP allows real-time monitoring of graft hemodynamic and metabolic parameters and timely identification of potentially unsuitable hearts [7].

The aim of the present study was to investigate the clinical outcomes of HTx in high-risk recipients who were transplanted only with grafts, selected according to extended donor criteria and preserved with EVP.

## Patients and Methods

### Study Population

All consecutive high-risk patients who underwent HTx with grafts from marginal donors and preserved with EVP at the University Hospital of Udine and Hannover Medical School, from 2016 to 2021, were retrospectively analyzed. Indication for EVP was the same in the two centers and involved in all cases employment of a marginal graft for a high-risk recipient, as subsequently defined. The study was approved by the Institutional Review Board (Clinical Registration Number: 18386, 1 August 2016) and informed consent was waived due to its retrospective nature. The major end-point of the study was the assessment of early and mid-term clinical outcome in terms of survival and major complications after HTx.

### Donor Heart Preservation

In the present series EVP was achieved in both participating centers employing the Organ Care System (OCS) (Transmedics Inc., Boston, MA, United States). The OCS perfusion technique has been described in detail previously [8]; briefly, it is instituted by cannulating the aorta and pulmonary artery of the graft and connecting them to a perfusion circuit with an oxygenator and a pulsatile pump. The beating heart is perfused with warm, oxygenated, nutrient-enriched donor blood mixed with priming solution. Graft function is assessed by continuous monitoring of aortic pressure, coronary flow and lactate trend. Final arterial lactate value (<5 mmol/L), lactate trend, difference between arterial and venous lactate concentration, visual contractility and stability of the perfusion data on OCS are all considered for HTx acceptance of donor grafts. The OCS device was transported either by car, plane or helicopter based on the distance of the donor hospital.

### HTx Procedure

Criteria for recipient selection and donor-recipient matching were based on standard guidelines [9–12]. HTx was performed using the bicaval anastomosis technique and immunosuppression therapy was standardized with steroids, calcineurin inhibitors and mycophenolate mofetil. Postoperative and long-term follow-up protocols at the University Hospital of Udine have been previously published and have remained unchanged during the study period [13–17]. In Hannover, all HTx recipients underwent triple immunosuppressive therapy with tacrolimus, prednisolone and mycophenolate mofetil. All patients underwent induction with anti-thymocite globulin on day 3, 4 and 5 after HTx. Endomyocardial biopsies were performed every 2 weeks during the first 3 months of follow-up. Seven patients who showed pre-transplant allosensitization and a positive virtual crossmatch were treated with a perioperative combination of therapeutic plasmapheresis, followed by infusions of Tocilizumab before allograft reperfusion, a single infusion of human immunoglobulins with or without a single infusion of Rituximab.

### Definition of Terms

High-risk recipients were defined as those on pre-HTx dependence from inotropic support, pre-HTx implantation of an intra-aortic balloon pump, those bridged to HTx with extracorporeal life support systems (ECLS) or a ventricular assist device (VAD). For ECLS support a Quadrox-i oxygenator and a Cardiohelp centrifugal pump (Getinge, Göteborg, Sweden), were employed. ECLS was used in 26% of patients in one center and in 5% in the other depending on different treatment policies of moderate primary graft dysfunction (PGD). Donors selected according to extended criteria, defined as “marginal,” were considered those ≥55 years of age, with a history of drug abuse, cardiac resuscitation or severe prolonged hypotension (>20 min), coronary artery disease, with at least a >30% stenosis of a major coronary branch, expected graft ischemia time ≥4 h, left ventricular ejection fraction <50%, interventricular septum thickness >14 mm, or body surface area difference between donor and recipient >20%. Hemodynamic support of the donor grafts was generally obtained with infusion of ≤0.1 y/kg/min of norepinephrine.

Chronic renal failure was defined by a glomerular filtration rate <50 mL/min/m^2^ or a persistent 50% increase of serum creatinine level. Impaired liver function was considered as at least a twofold increase of bilirubin and/or liver enzymes.

PGD was defined according to the International Society for Heart and Lung Transplantation (ISHLT) consensus statement, considering it relevant when grade ≥ moderate [18, 19].

Acute renal failure was defined following the Risk of renal dysfunction, Injury to the kidney, Failure of kidney function, Loss of kidney function, End-stage kidney disease (RIFLE) criteria [20]. The indication for continuous renal replacement therapy was based on persistent oliguria (<0.5 mL/Kg/h) for at least 6 h, a double increase of serum creatinine levels or at least 50% reduction of the glomerular filtration rate within 24 h.

Infections were defined as any episode requiring antibiotic, antiviral or antifungal treatment. Acute rejection was diagnosed, scored and treated following the ISHLT guidelines [21]. Grade ≥ 2 acute rejection or any type of rejection, cellular and/or antibody mediated with hemodynamic impairment was considered as a post-HTx complication and treated accordingly. Coronary allograft vasculopathy was diagnosed by yearly angiographies and defined according to ISHLT classification [22, 23].

For prediction of PGD, the RADIAL score has been used which is calculated on six factors with similar influence, four of which are related to the recipient: Right atrial pressure ≥10 mmHg, Age ≥60 years, Diabetes and Inotropic support dependence while two are related to the donor: Age ≥30 years and Length of total graft ischemic time ≥240 min. The presence of each of these factors in an individual patient adds 1 point to the final PGD predictive score [6].

### Statistical Analysis

Continuous variables were expressed as mean ± standard deviation or median and range (min-max) according to the data distribution, after performing the Kolmogorov-Smirnov test for normality. Categorical variables were presented as absolute numbers and percentages. Cumulative overall survival was defined as freedom form all-cause mortality and was determined by the Kaplan-Meier method. Binary logistic regression was used to assess factors for PGD ≥ moderate and in-hospital mortality, while Cox-regression model was used for long-term mortality after HTx. De Long’s nonparametric receiver operating characteristic analysis of the area under the curve (AUC) was performed to estimate the accuracy of risk factors that were identified at the univariate analysis and to determine a cut-off value.

Analyses were performed with IBM SPSS Statistics 22 for Microsoft Windows (IBM Corp., Armonk, NY).

## Results

### Baseline Characteristics

During the study period, out of 88 marginal grafts 80 were employed for HTx in high-risk recipients; 37% were transplanted at the University Hospital of Udine and 71% at the Hannover medical School; 8 (10%) were discarded after being considered unsuitable for HTx through OCS graft assessment because of pathological increase of lactates despite adequate coronary perfusion in most cases; in particular, right ventricle dysfunction, severe aortic valve regurgitation, coronary anomaly and coronary dissection were detected in four grafts.

### Patients Data

Recipient data are shown in Table 1. Median age was 57 years (range, 13–75); 80% were males, with a mean body mass index of 26 ± 4 and 12 patients had a weight ≥100 kg and a body mass index ≥30 with a significant size mismatch occurring in 30% of them. Chronic renal failure was present in 61% patients, three of whom where in pre-HTx dialysis and underwent combined heart-kidney transplantation; 11% had impaired liver function and 90% had a previous cardiac operation; 66% were bridged to HTx on long-term VAD with a median support time of 22 months (range, 1–133). In 81% urgent HTx was performed: of these 14% were on short-term ECLS (mean support time of 12 ± 12 days), 12% were dependent from inotropic support and 55% were patients with VAD-related complications. Median RADIAL score was 3 (range, 0–6); a score ≥4 was present in 35% of recipients.

**TABLE 1:** Patients data.

	*N* = 80
Median age, years (range)	57 (13–75)
Male sex, n (%)	64 (80)
Mean BMI	26 ± 4
Diabetes, n (%)	7 (9)
Mean serum creatinine (mg/dL)^a^	1.68 ± 0.58
Median GFR, mL/min/m^2^, (range)^a^	38 (16–82)
Median SGOT, UI/L (range)	35 (11–110)
Mean SGPT, UI/L	37 ± 16
Median bilirubin, mg/dL (range)	1.22 (0.29–6.47)
Mean PAP, mmHg	38 ± 13
Median CVP, mmHg (range)	12 (2–28)
Previous cardiac surgery, n (%)	72 (90)
Long-term VAD, n (%)	53 (66)
ECLS, n (%)	11 (14)
Inotropic dependence, n (%)	9 (12)
Urgent HTx, n (%)	65 (81)
Median RADIAL score (range)	3 (0–6)

BMI, body mass index; GFR, Glomerular filtration rate; SGOT, Serum glutamic-oxaloacetic transaminase; SGPT, serum glutamate pyruvate transaminase; INR, international normalized ratio; PAP, systolic pulmonary artery pressure; CVP, central venous pressure; VAD, ventricular assist device; HTx, Heart transplantation; ECLS, Extra-corporeal life support.

^a^ Values reported exclude patients having combined heart and kidney transplantation.

### Donor Data

Donor data are summarized in Table 2. Median donor age was 48 years (range, 17–69); 28% were ≥55 years of age, 65% were males, 46% smokers, 29% had a history of alcohol abuse and 39% suffered a previous episode of cardiac arrest. On transthoracic 2D- echo, 6% of grafts had a left ventricular ejection fraction ≤50%, while 21% showed left ventricular hypertrophy; in 4% coronary artery disease was disclosed at angiography revealing 40% stenosis of the left anterior descending in two cases and stenoses of 45% and 50% of left anterior descending and right coronary artery in another, respectively.

**TABLE 2:** Donor data.

	*N* = 80
Median age, years (range)	48 (17–69)
Age ≥55 years, n (%)	22 (28)
Male sex, n (%)	52 (65)
LVEF ≤50%, n (%)	5 (6)
Mean LVEF, %	56 ± 10
Mean LV diastolic diameter, cm	4.6 ± 0.5
Mean LV systolic diameter, cm	3.4 ± 1.3
Median IVS, mm (range)	12 (7–16)
LV hypertrophy, n (%)	17 (21)
Cardiac arrest/prolonged severe hypotension, n (%)	31 (39)
Mean CPR time, min	28 ± 15
CAD, n (%)	3 (4)
Diabetes, n (%)	1 (1)
Smoking habit, n (%)	37 (46)
Alcohol abuse, n (%)	23 (29)

LVEF, left ventricular ejection fraction; LV, left ventricle; IVS, interventricular septum; CPR, Cardio-pulmonary resuscitation; CAD, coronary artery disease.

### Early Outcomes

Mean graft ischemic time was 118 ± 25 min, mean EVP time 289 ± 62 min, mean total “out-of-body” time 420 ± 66 min and median cardiopulmonary bypass (CPB) time 228 min (range 126–416 min). Three patients underwent combined heart- kidney transplantation (Table 3).

**TABLE 3:** Intra-operative data.

	*N* = 80
Mean EVP time, min	289 ± 62
Mean ischemic time, min	118 ± 25
Mean out of body time, min	420 ± 66
Median CPB time, min (range)	228 (126–416)
Combined heart-kidney transplantation	3

EVP, *Ex-vivo* perfusion; CPB, Cardio-pulmonary by-pass.

Post-operative data are shown in Table 4. In-hospital mortality was 11%. Causes of early deaths were septic (*n* = 2) or haemorrhagic shock (*n* = 2), pancreatitis (*n* = 2), multi-organ failure (*n* = 2), and ECLS-related complications (*n* = 1). Postoperatively, 15 patients (19%) needed *de novo* ECLS support, because of ≥ moderate PGD in 13 patients (16%), vasoplegia syndrome in 1 and respiratory insufficiency in 1. Moreover, two patients needed veno-venous ECLS for pulmonary complications while an intra-aortic balloon was implanted in three patients. Two patients were assisted with ECLS for <24 h and 8 for <72 h.

**TABLE 4:** Post-operative data.

	*N* = 80
Overall complications, n (%)	69 (86)
Moderate/severe PGD, n (%)	13 (16)
ECLS, n (%)	17 (21)
Pre-Htx ECLS	2
IABP, n (%)	3 (4)
Median MAV time, hours (range)	29 (3–2,649)
MAV >72 h, n (%)	21 (26)
Tracheostomy, n (%)	14 (18)
Revision for bleeding, n (%)	15 (19)
Need for CRRT, n (%)	51 (64)
Stroke, n (%)	1 (1)
Atrial fibrillation, n (%)	19 (24)
Median ICU stay (days, range)	6 (1–123)
Median hospital stay (days, range)	36 (3–236)
In-hospital mortality, n (%)	9 (11)

PGD, primary graft dysfunction; ECLS, extra corporeal life support; IABP, Intra-aortic balloon pump; MAV, mechanical assisted ventilation; CRRT, continuous renal replacement therapy; ICU, intensive care unit.

Median intensive care unit stay was 6 days (range, 1–123); median mechanical assisted ventilation time was 29 h (range, 3 h to 110 days) with 26% of patients requiring >72 h of ventilation and 18% a tracheostomy. Median hospital stay was 36 days (range, 3–236), during which 64% of patients needed dialysis for acute renal failure, 24% had new onset of atrial fibrillation and 11% acute rejection grade ≥2 (combined with antibody-mediated rejection 2 in two cases); 19% needed sternal re-entry for bleeding and 1% had a stroke.

At univariable analysis, CPB time resulted a risk factor for both ≥moderate PGD (*p* = 0.001) and in-hospital mortality (*p* = 0.031).

In addition, high pre-HTx central venous pressure (CVP) was also a risk factor for hospital mortality (*p* = 0.050); PGD ≥ moderate and in-hospital mortality predictions of the CPB time showed AUC of 0.82 and 0.73 with cutoff values of 246 and 272 min, respectively. The predictive role of CVP regarding in-hospital mortality showed an AUC of 0.69, with a cutoff value of 15 mmHg (Table 5).

**TABLE 5:** Results of univariable analysis.

	Odds ratio	95% CIs	*p*-value
Risk factors for ≥moderate PGD			
CPB time	1.019	1.007–1.032	0.001
Risk factors for in-hospital mortality			
CPB time	1.001	1.001–1.023	0.031
Pre-HTx CVP	1.155	1.000–1.333	0.050
Risk factors for late mortality	Hazard ratio	95% CIs	*p*-value
Recipient age	6.619	1.331–32.904	0.021
Pre-HTx ECLS	6.183	1.542–24.798	0.010
Donor age	1.089	1.088–1.177	0.031

PGD, primary graft dysfunction; CPB, cardiopulmonary bypass; HTx, Heart transplantation; CVP, central venous pressure; ECLS, extracorporeal life support; IABP, Intra-aortic balloon pump.

### Follow-Up Data

**FIGURE 1:**
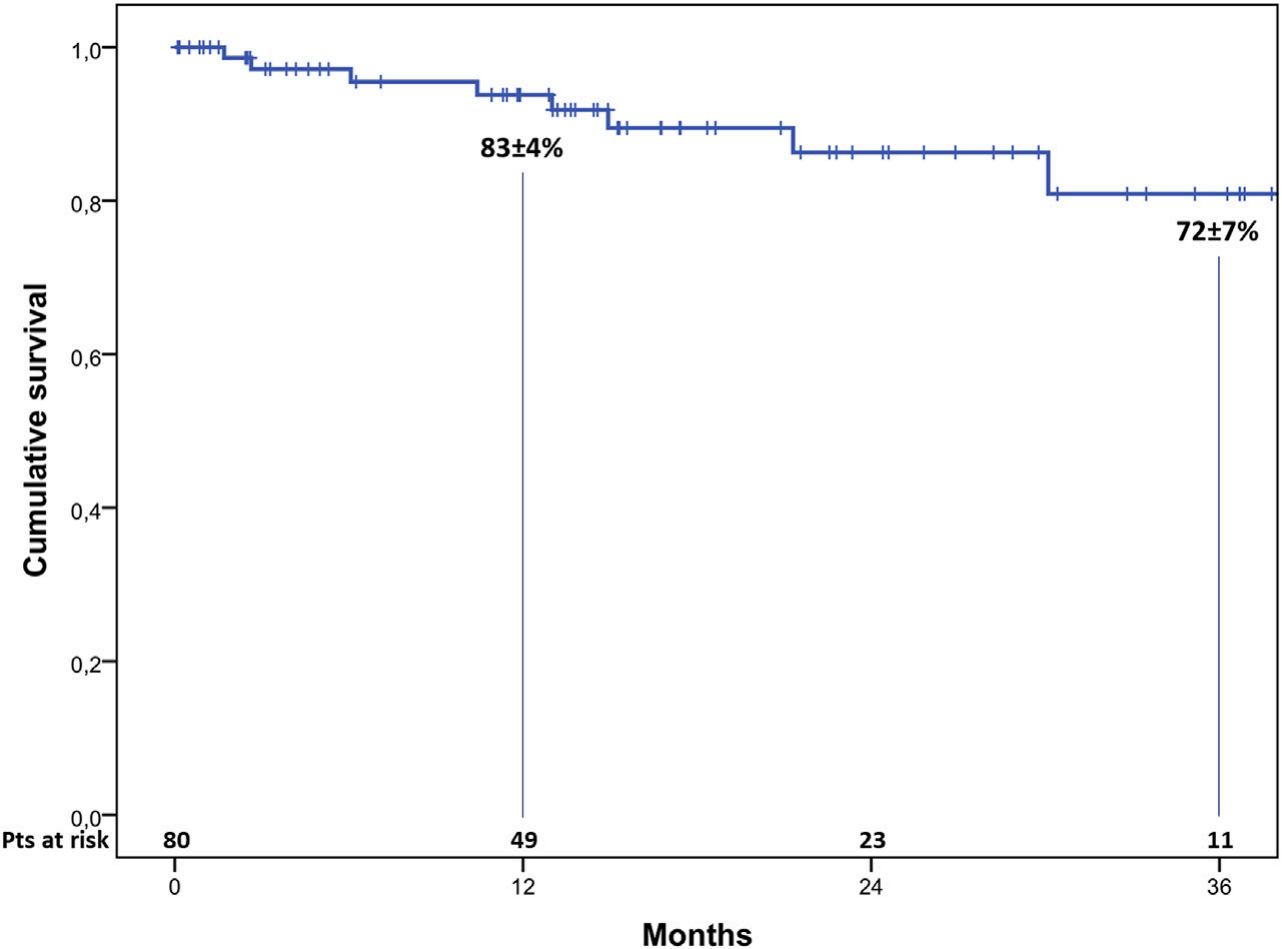
Actuarial 3-year survival after heart transplantation.

During a median follow-up of 16 months (range, 2–43 months), eight patients died. The causes of late mortality are shown in Table 6. Actuarial survival at 1 and 3 years HTx was 83% ± 4% and 72% ± 7% (Figure 1). The rate of grade ≥2R acute rejection episodes and coronary allograft vasculopathy during follow-up was 6% and 10%, respectively.

**TABLE 6:** Long-term complications.

	Survivors *n* = 71
Median follow-up, months (range)	16 (2–43)
Rejection grade ≥2R, n (%)	5 (6)
CAV, n (%)	7 (10)
Late mortality, n (%)	8 (10)
Cardiac	2
Infection	3
Stroke	1
Cerebral hemorrhage	1
Neoplasia	1

CAV, coronary allograft vasculopathy.

At univariable analysis, recipient age (*p* = 0.021), pre-HTx ECLS (*p* = 0.010), donor age (*p* = 0.031) were reported to be risk-factors for late mortality (Table 5).

## Discussion

HTx represents the gold standard surgical treatment of end-stage heart failure with improved early and late post-HTx outcomes; however, the rate of PGD continues to be relatively high [8]. The interaction of donor, recipient and procedural factors may predispose to this life-threatening complication which represents the leading cause of 30-day mortality post-HTx [9]. The improvement of medical and interventional therapies, as well as the widespread use of MCS systems, have led to consider as potential candidates for HTx a subset of patients with end-stage heart failure with increasing age and multiple comorbidities. Based on the data reported by the ISHLT registry in 2019, in the last decade, 50% of HTx recipients have had a prior cardiac surgery, 39% were dependent on inotropic support and 5% had history of dialysis before HTx [10]. In the present series, 90% of recipients had a previous cardiac surgery, 61% chronic renal failure and 12% were on inotropic support, while three patients in dialysis pre-HTx underwent a combined heart and kidney transplantation. Therefore, these recipients were considered at high risk of developing PGD, 32% of them having a RADIAL score ≥ 4. In this study we have used the RADIAL score to predict post-HTx PGD since we believe it a simple, still reliable and easy-to-use tool particularly when data collection involves International centers; other scores, reported to have a more predictive accuracy, either consider a large variety of factors, including many intraoperative data, which were not available for our analysis, or analyzes only the recipient-related risk factors [20, 21].

Among high-risk patients a specific group that could particularly benefit of EVP are those requiring MCS as a bridge to HTx [14]. In this series the rate of patients on MCS was much higher than that reported in the ISHLT registry, 66% being on VAD and 14% on ECLS. Although currently long-term VAD recipients are not uniformly considered as high-risk patients, data from the European Registry of Mechanical Circulatory Support (EUROMACS) recognize VAD as a risk factor for post-HTx mortality as also confirmed by some reports from the United States [24, 25]. Particularly, among our patients many required urgent HTx because of life threatening complications related to the long-term VAD implantation. HTx in patients after long-term assistance with VAD is more complex and technically demanding, due to the presence of coarse adhesions and bleeding due to anticoagulation which often require prolonged CPB times. In the present study a longer CPB time, generally required for more complex redo procedures such as those in patients with VAD, has been found to be a risk factor at univariable analysis for both hospital mortality and PGD as also confirmed by others [19]. Similar problems have been encountered also in ECLS bridged patients, due to their generally more critical hemodynamic conditions and frequent multiorgan impairment. Furthermore, also a high CVP has been found to be a risk factor for early death as also recognized in the RADIAL score system; indeed, the effects of a high CVP, mainly on the splancnic district, are well known, conditioning the patient status pre-HTx and thus the outcome [8].

Considering the chronic donor shortage, patients at higher risk and with multiple comorbidities generally have a reduced probability to be considered as suitable HTx candidates. Similarly, also obese patients, who are more likely to have a significant donor/recipient size mismatch, may have less chances to be transplanted; moreover, size mismatch becomes a significant risk factor particularly when associated to other high-risk characteristics of both recipients or donors [12, 26]. In our study 30% of patients had a significant donor/recipient size mismatch; however, this data has not been analyzed separately because of the small number of cases generally associated with other important risk factors.

To more effectively address the issue of organ shortage, donor criteria have been extended, enlarging the available donor pool, but potentially also increasing the risk of adversely affecting the outcomes after HTx by employing suboptimal grafts. In performing a donor risk analysis, the use of the Eurotransplant donor heart risk score has been suggested to facilitate donor risk assessment allowing for more appropriate matching of extended criteria donor hearts [27]. However, it does not include among the donor factors considered the ischemia time, which is one of the most important variables, the one we actually tried to restrain with the use of OCS. Furthermore, the variables included in our analysis are those commonly used in other similar studies including the more recent Expand trial [28].

In the effort to increase the donor pool by using also marginal donors, alternative techniques of graft protection, such as the OCS, have been suggested yielding gratifying results [13]. It has been demonstrated that the time-dependent negative impact of ischemia on graft function depends on the donor age since prolonged ischemia is poorly tolerated by grafts from older donors [29]. Indeed, EVP provides a better myocardial protection, not only by allowing to limit the graft ischemic time, but also to assess and potentially recondition the donor heart [13–17]. Analysis of histological biopsy samples confirms that cardiomyocyte damage was either stable or even improved after reperfusion following HTx in EVP supported hearts, while after cold storage preservation donor grafts showed at histology worsening of myocardial damage after reperfusion [12]. Cardiomyocyte degeneration and edema increased after 6 h of support and, therefore, OCS perfusion longer than 8 h should be avoided [17]. This is supported by others who demonstrated that the length of time on OCS was a strong predictor of PGD [19]. Thus, employment of the OCS, the only device currently available for normothermic *ex-vivo* heart perfusion, could have an important role in graft protection limiting the rate of PGD and PGD-related mortality, especially when critical recipients receive hearts from high-risk donors [13, 30–34].

Morbidity and mortality of the present experience are worse than those previously observed in both Institutions after HTx using standard donors in low risk recipients [35, 36]; however, they are quite comparable to those previously reported by García Sáez et al. who observed excellent short-term outcomes with the OCS in high-risk patient transplanted with marginal donors, despite the higher age of donors in our series [30, 35, 36]. Our results are also similar to those reported by the ISHLT registry [12] and may be explained not only by reduction of the ischemic times and better graft preservation, but also by the possibility of identifying, during EVP, unsuitable marginal grafts [7, 15]. Indeed, in the present experience 10% of donor grafts were discarded because timely detection of cardiac pathologies diagnosed owing to the OCS system.

Despite good early survival many patients experienced postoperative complications with a consequently extended intensive care unit stay. An increased need for chest re-entry for postoperative bleeding was observed mainly in patients undergoing HTx on VAD or reoperations, frequently associated to coagulation disorders. Also a high rate of renal failure and respiratory insufficiency requiring dialysis or prolonged mechanical ventilation was observed. It must be underlined that the high incidence of dialysis reflects the policy of one of the two centers, which in case of postoperative worsening of kidney function preferred to sustain it with early replacement therapy. Furthermore, the high number of patients requiring post-HTx ECLS implantation (21%) is related to a more liberal use of ECLS owing to the greater safety of such systems in the current era. This reflects differences in the centers policy, since one Institution preferred to employ early ECLS for a limited time, generally from <24 to 72 h, in patients with signs predicting the possible onset of even moderate PGD. The rate of PGD in our study was quite acceptable, being 16%, not much higher than what reported in the Expand trial [28] and by García Sáez et al [30], despite the different profiles of patients analyzed in such studies, regular recipients in the first and younger donor age in the second.

### Study Limitations

This study has certainly some limitations mostly represented by its retrospective nature and those pertaining to any multicenter collaboration. In this specific study only two centers were involved minimizing potential biases in patient selection and treatment; however, some differences in each center policy, especially in postoperative immunosuppressive treatment and follow-up protocols, have emerged, which might have had an impact on patient outcomes but with negligible influence on PGD and perioperative complications. Moreover, the number of patients enrolled in this study was limited due to the specific patient characteristics, both of donor and recipients, thus precluding to select a control group for comparison.

### Conclusion

Our study, dealing with a very complex setting represented by marginal donors, very high-risk recipients and expected long ischemic time, indicates that EVP appears to be an effective tool in reducing overall donor graft ischemic time and allowing continuous evaluation of graft function and viability during transportation. This should provide adequate grafts for high-risk recipients who would be otherwise excluded from the possibility of a HTx. Nevertheless, based on the gratifying results observed in the present study, we advocate the employment of EVP using OCS technology as a promising and valid tool to further extend the donor pool, to successfully perform HTx even in high-risk settings.

## Data Availability

The raw data supporting the conclusion of this article will be made available by the authors, without undue reservation.

## Abbreviations

CPB, cardio-pulmonary bypass; ECLS, extra-corporeal life support; EVP, *ex-vivo* perfusion; HTx, heart transplantation; MCS, mechanical circulatory support; OCS, Organ Care System™; PGD, primary graft dysfunction; VAD, ventricular assist device; ISHLT, International Society for Heart amd Lung Transplantation.
